# Mortality of Nonoperatively Managed Geriatric Pelvic Ring Fractures

**DOI:** 10.7759/cureus.105712

**Published:** 2026-03-23

**Authors:** Devon R Pekas, Alexander R Garcia, Youngjae Lee, Franco M Coniglione, Cody L Evans, Jesse B Seamon

**Affiliations:** 1 Department of Orthopedic Surgery, Carilion Clinic, Roanoke, USA

**Keywords:** fragility fracture, geriatric pelvic ring fracture, matched cohort, mortality, nonoperative management

## Abstract

Objective

The purpose of this investigation was to identify the mortality rate at three months and one year for patients 65 years old or older with a nonoperatively managed pelvic ring fracture.

Materials and methods

A retrospective comparative cohort study was performed at a single level 1 trauma tertiary referral center. From 2014 to 2019, we reviewed all patients older than 65 years who underwent nonoperative treatment for a pelvic ring fracture (Current Procedural Terminology codes 27193 or 27197). Demographics, including age, sex, body mass index (BMI), and Charlson Comorbidity Index (CCI), were recorded. Mortality status and death dates were determined from our institutional electronic health record and cross-referenced with the TriNetX database (Cambridge, MA, USA). The primary outcome was the standardized mortality ratio (SMR) for one-year mortality, calculated by comparing observed deaths in the study population with expected deaths in the age- and sex-matched general population. Secondary outcomes included three-month and one-year mortality rates, as well as demographic variables associated with mortality.

Results

A total of 307 patients met the inclusion criteria. There were 41 (13.4%) that died within three months and 77 (25.1%) that died within 1 year. Compared to the general population, pelvic ring fracture patients demonstrated an SMR of 3.12 (95% confidence interval: 2.46, 3.85) for one-year mortality. Compared with patients alive at three months post-injury, deceased patients did not differ significantly in age (80.9 ± 8.6 vs. 81.0 ± 9.7 years; p = 0.964), BMI (24.3 ± 5.2 vs. 25.9 ± 5.9; p = 0.062), or sex (p = 0.507). Alive patients had a lower mean CCI compared to deceased patients (5.4 ± 2.1 vs. 6.8 ± 2.8; p < 0.001). At one year, deceased patients did not demonstrate statistically significant age differences (80.5 ± 8.5 vs. 82.4 ± 9.5 years; p = 0.103), BMI (24.5 ± 5.3 vs. 24.5 ± 5.5; p = 0.922), or sex (p = 0.835), and alive patients had a lower mean CCI (5.2 ± 2.0 vs. 6.6 ± 2.6; p < 0.001).

Conclusions

Geriatric patients who undergo nonoperative treatment of pelvic ring fractures have a threefold higher one-year mortality compared to their age- and sex-matched peers who do not have pelvic ring fractures. There is an association between mortality and higher comorbidity burden, and clinicians should counsel patients on the elevated risk of post-injury mortality based on comorbidity status. This has implications for counseling patients and their families, as such an approach may inform surgical decision-making and ensure that treatment plans are tailored to the patient's overall health and prognosis.

## Introduction

Pelvic ring fractures consist of combined fractures of the innominate bones and sacrum with ligamentous injuries involving the sacroiliac, sacrotuberous, and sacrospinous ligaments. Geriatric pelvic ring fractures have been characterized as fragility fractures (i.e., low-energy fractures in patients with abnormal underlying bone due to medical comorbidities). A hallmark characteristic of fragility fractures is that they are often sentinel events of impending medical decline and are associated with increased mortality [1,2]. The annual incidence of pelvic ring fractures is 36 per 100,000 patients in the geriatric population [3-5], and it is important to be aware of the mortality risk following these fractures to be able to adequately counsel patients and their families.

Prior studies have demonstrated that pelvic ring fractures are associated with high mortality rates ranging from 12% to 39% at one year [3,6-10]. While the absolute mortality rates have been reported, it is still not well understood whether geriatric patients with pelvic ring fractures demonstrate increased mortality risk relative to their peers. The measurement that quantifies this relative mortality is known as the standardized mortality ratio (SMR), and this has become a helpful metric for assessing prognosis and mortality risk following various fragility fractures [11].

Another limitation of existing studies is that they often combine nonoperatively and operatively treated patients in their analysis. Operative treatment may alter the natural history of this injury and be associated with reduced mortality, although this remains under investigation [6,7,12]. Additionally, operatively treated patients represent a more heterogeneous group, comprising both low-energy fragility fractures and high-energy fractures. High-energy fractures represent a fundamentally different injury and more commonly occur in younger patients with a different risk profile. High-energy fractures are also more likely to be unstable and meet operative indications [13]. In this preliminary study, we focused exclusively on nonoperatively treated patients, as they predominantly represent stable geriatric fragility fractures.

The findings of this study will help the field better understand the mortality risk associated with fragility fractures of the pelvic ring and comment on how they compare to mortality risk profiles of other fragility fractures (e.g., hip fractures). We hypothesize that mortality rates will be similar between our cohort of geriatric pelvic ring fractures and prior estimates for geriatric hip fractures. For ≥65-year-old patients with nonoperatively managed pelvic ring fractures, our primary aim was to determine one-year SMR relative to the age- and sex-matched U.S. general population. Secondary aims included evaluating three-month and one-year mortality rates in our institutional cohort and determining patient-specific demographic variables associated with increased mortality.

## Materials and methods

Study design and cohort

After institutional review board approval, we queried a single level 1 trauma center’s electronic health record (EHR) for geriatric patients who had a nonoperatively managed pelvic ring fracture between January 1, 2014, and December 31, 2019. Inclusion criteria were age ≥65 years and nonoperative management of a pelvic ring fracture, as determined by Current Procedural Terminology (CPT) codes 27193 or 27197. Patients with pelvic ring fractures and concurrent sacral fractures were included. Exclusion criteria were concurrent femur fracture or incomplete records. While we did not explicitly exclude unstable fracture patterns, patients at our institution with unstable fractures are predominantly treated operatively. Patients with operatively treated fractures were not included in the study.

Institutional protocol for management of geriatric pelvic ring fractures

Given the current lack of consensus on the definition of acceptable alignment and indications for operative treatment [14], we have developed an institutional protocol for the management of geriatric pelvic ring fractures without major displacement on initial injury radiographs, for which the treating attending surgeon deems the pattern stable. This protocol includes a trial of ambulation, pain management, and physical therapy (PT). Patients are allowed to work with PT on transfers and ambulation following the decision to pursue nonoperative management. Patients who have unstable fractures, fail to mobilize, or have significant pain following a trial of nonoperative management are generally treated operatively.

Data extraction

Manual record review was performed to assess the mortality status for our geriatric pelvic ring fracture patients and, if applicable, the date of death for all deceased patients. These were determined from our institutional EHR and cross-referenced with TriNetX (Cambridge, MA, USA), a national database that aggregates records from multiple healthcare organizations. Patients without death dates recorded in either our institutional records or TriNetX were considered alive in our analysis, and deaths recorded at non-TriNetX institutions were not captured. Patients with death dates recorded in either our institutional records or TriNetX were considered deceased. The starting time for mortality analysis was set to the treatment date, as determined by the CPT code, at the first point of patient contact upon presentation to the emergency department or clinic. Demographic data, including age, sex, body mass index (BMI), and the Charlson Comorbidity Index (CCI), were retrieved for each patient. The CCI is a composite score ranging from 0 to 37 points, based on the presence of the following advanced conditions: cardiovascular, pulmonary, gastrointestinal, neurologic, and hematologic/oncologic [15].

Data analysis

The primary outcome of interest was the SMR, defined as the ratio of observed deaths in our study population to expected deaths in the age- and sex-matched general population [16]. Expected deaths in the general population were calculated based on survival probabilities reported in the U.S. Social Security Administration's 2021 Period Life Table [17]. Secondary outcomes included three-month and one-year mortality rates. For each demographic variable, descriptive statistics were reported, and univariate logistic regression was performed to estimate the odds ratio for three-month and one-year mortality. All statistical analyses were performed using RStudio (2024.04.2 Build 764, Posit PBC, Boston, MA, USA) with a significance level of 0.05.

## Results

**Figure 1:**
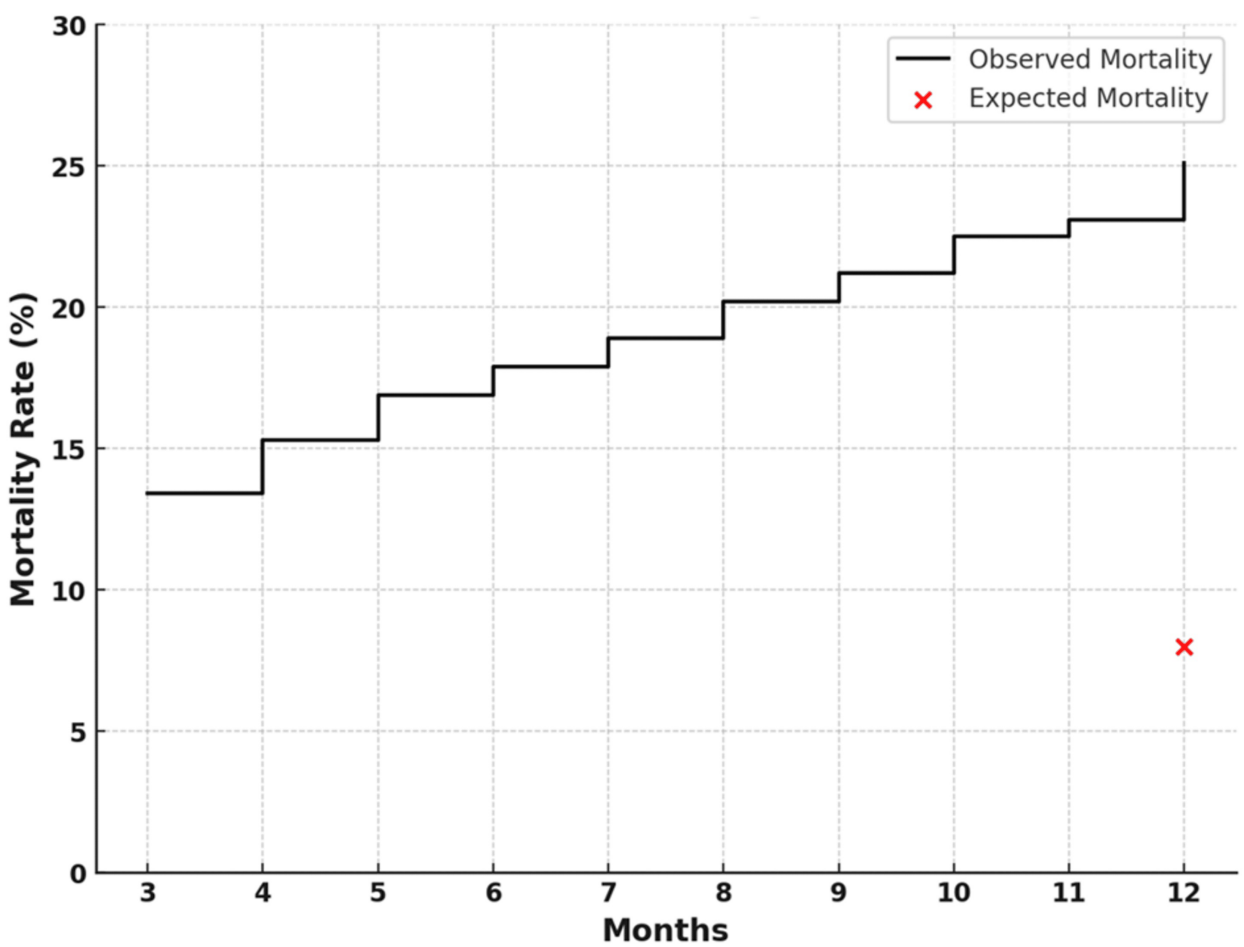
Mortality following nonoperatively managed geriatric pelvic ring fractures The solid black line indicates the observed mortality rate in the study population over time. The red "X" indicates the expected one-year mortality rate of the age and sex-matched general population. Image Credit: Authors using PowerPoint (Microsoft Corp., Redmond, WA, USA)

A total of 307 patients met the inclusion criteria. 41 (13.4%) died within three months, and 77 (25.1%) died within one year (Figure 1). The expected mortality for the age- and sex-matched general population was 24.7 (8.0%) within one year (Figure 1). Compared with the general population, our study population of nonoperatively managed geriatric patients with pelvic ring fractures had an SMR of 3.12 (95% confidence interval: 2.46, 3.85) for one-year mortality.

Compared to alive patients, patients who were deceased at three months post-treatment did not have statistically significant age differences (80.9 ± 8.6 vs. 81.0 ± 9.7 years; p = 0.964), BMI (24.3 ± 5.2 vs. 25.9 ± 5.9; p = 0.062), or sex differences (p = 0.507; Table 1). Alive patients had a lower mean CCI score compared to deceased patients (5.4 ± 2.1 vs. 6.8 ± 2.8; p < 0.001; Table 1).

**Table 1:** Demographic variables associated with three-month mortality * Statistically significant result. Based on the results of univariate logistic regression, odds ratios were calculated to measure the association between each demographic variable and three-month mortality. Continuous variables are represented as mean ± standard deviation, and categorical variables are represented as counts with percentages. BMI: body mass index, CCI: Charlson Comorbidity Index, CI: confidence interval, OR: odds ratio

Variable	Alive	Deceased	OR (95% CI)	p-value
N (%)	266 (86.6)	41 (13.4)	-	-
Age (years)	80.9 ± 8.6	81.0 ± 9.7	1.00 (0.96, 1.04)	0.964
BMI (kg/m^2^)	24.3 ± 5.2	25.9 ± 5.9	1.06 (1.00, 1.12)	0.062
Sex (% male)	65 (24.4%)	12 (29.3%)	1.28 (0.60, 2.60)	0.507
CCI	5.4 ± 2.1	6.8 ± 2.8	1.27 (1.11, 1.45)	<0.001*

Compared to alive patients, patients who were deceased at one year post-treatment did not have statistically significant age differences (80.5 ± 8.5 vs. 82.4 ± 9.5 years; p = 0.103), BMI (24.5 ± 5.3 vs. 24.5 ± 5.5; p = 0.922), or sex differences (p = 0.835; Table 2). Alive patients had a lower mean CCI score compared to deceased patients (5.2 ± 2.0 vs. 6.6 ± 2.6; p < 0.001; Table 2).

**Table 2:** Demographic variables associated with one-year mortality * Statistically significant result. Based on the results of univariate logistic regression, odds ratios were calculated to measure the association between each demographic variable and one-year mortality. Continuous variables are represented as mean ± standard deviation, and categorical variables are represented as counts with percentages. BMI: body mass index, CCI: Charlson Comorbidity Index, CI: confidence interval, OR: odds ratio

Variable	Alive	Deceased	OR (95% CI)	p-value
N (%)	230 (74.9)	77 (25.1)	-	-
Age (years)	80.5 ± 8.5	82.4 ± 9.5	1.03 (1.00, 1.06)	0.103
BMI (kg/m^2^)	24.5 ± 5.3	24.5 ± 5.5	1.00 (0.95, 1.05)	0.922
Sex (% male)	57 (24.8%)	20 (26.0%)	1.06 (0.58, 1.90)	0.835
CCI	5.2 ± 2.0	6.6 ± 2.6	1.31 (1.16, 1.48)	<0.001*

## Discussion

This study identifies an association between the presence of a geriatric pelvic ring fracture treated nonoperatively and a threefold increased risk of one-year mortality compared to age and sex-matched peers. Our results demonstrate that mortality rates remain high, with 13.4% at three months and 24.1% at one year post-treatment. These findings are comparable to prior literature reporting 12-39% one-year mortality rates for geriatric pelvic ring fractures, highlighting the frailty of this population and impending decline following this injury [3,6-10]. Both the one-year SMR and one-year mortality rate for geriatric pelvic ring fractures are comparable with historical estimates for geriatric hip fractures [18]. Notably, this study included a larger patient cohort than previously reported studies [3-5,8-10].

Comorbidity status was also linked to elevated risk of mortality at both three months and one year. Prior studies have demonstrated that the cause of death for the majority of geriatric patients after pelvic ring fracture is cardiovascular disease [2]. Early mobilization and optimization of medical comorbidities are critical to improve outcomes. Yet, the lack of universal guidelines for treating these fractures leaves clinicians reliant on individualized, case-specific decision-making [3,6,7,19-21]. Certain studies suggest that patients treated with internal fixation may have decreased mortality compared to those who receive nonoperative treatment [22]; however, it is unclear whether patients treated nonoperatively have more severe comorbidities.

The goal of this study was to examine mortality risk in nonoperatively treated geriatric pelvic ring fractures. While we did not review each patient's radiographs to determine whether the fracture pattern was stable, in general, stable fractures are treated nonoperatively at our institution. Although a small subset of patients with unstable fractures may have been treated nonoperatively due to comorbidities, we believe this is an uncommon scenario and that our sample predominantly represents stable geriatric pelvic ring fractures. Additionally, while the exact PT regimen patients underwent was tailored to their individual needs, all trauma surgeons within our institution adhered to our general treatment protocol of early ambulation, pain management, and PT. The goal of this study was to evaluate mortality in nonoperatively treated pelvic ring fractures rather than to assess the impact of our institutional treatment protocol, which is provided for additional context.

This investigation has several limitations. First, the accuracy of the mortality data is limited by the timing of data extraction from each database, as there is a delay between the patient's death and its official reporting. However, using the TriNetX database enabled us to capture additional deaths not reported in our institutional EHR. This enhanced the accuracy of the mortality data in this study compared with the existing literature. Another limitation is the inherent selection bias due to including only nonoperatively treated patients. This decision was made intentionally, given that the focus of the present study was to compare mortality in nonoperatively treated pelvic ring fracture patients to age- and sex-matched peers. Some existing literature suggests that operative treatment may alter the natural history and mortality risk associated with geriatric pelvic ring fractures [12]. In future studies, we plan to include an operative cohort to determine if operative treatment may reduce mortality risk.

Additionally, although univariate logistic regression was performed, multivariate logistic regression was not, as identifying factors associated with increased three-month and one-year mortality was a secondary aim of the present study. In future studies, we plan to perform multivariate analysis, adjusting for demographics and comorbidities, to identify factors independently associated with mortality risk. Lastly, other limitations include the absence of analysis by fracture pattern (i.e., Tile/Young-Burgess classification), fracture stability, functional outcomes, cause of death, anabolic therapy for osteoporosis, bone mineral density, frailty score, sarcopenia, cognitive impairment, mental health, and ambulation status, all of which may impact mortality. We were unable to control for these potential confounders in the present study, and we plan to investigate these factors in future studies.

## Conclusions

This study suggests high mortality rates following nonoperatively managed geriatric pelvic ring fractures. It also highlights the need for prospective research to better delineate the factors contributing to mortality and to evaluate the potential benefits of emerging treatment paradigms. The elevated risk of mortality after geriatric pelvic ring injuries should inform shared decision-making between clinicians, patients, and families, while also serving as a foundation for future work to refine the management of this growing public health challenge.
